# Navigation in Real-World Environments: New Opportunities Afforded by Advances in Mobile Brain Imaging

**DOI:** 10.3389/fnhum.2018.00361

**Published:** 2018-09-11

**Authors:** Joanne L. Park, Paul A. Dudchenko, David I. Donaldson

**Affiliations:** Department of Psychology, Faculty of Natural Sciences, University of Stirling, Stirling, United Kingdom

**Keywords:** spatial navigation, mobile brain imaging, virtual-reality (VR), EEG, fNIRS

## Abstract

A central question in neuroscience and psychology is how the mammalian brain represents the outside world and enables interaction with it. Significant progress on this question has been made in the domain of spatial cognition, where a consistent network of brain regions that represent external space has been identified in both humans and rodents. In rodents, much of the work to date has been done in situations where the animal is free to move about naturally. By contrast, the majority of work carried out to date in humans is static, due to limitations imposed by traditional laboratory based imaging techniques. In recent years, significant progress has been made in bridging the gap between animal and human work by employing virtual reality (VR) technology to simulate aspects of real-world navigation. Despite this progress, the VR studies often fail to fully simulate important aspects of real-world navigation, where information derived from self-motion is integrated with representations of environmental features and task goals. In the current review article, we provide a brief overview of animal and human imaging work to date, focusing on commonalties and differences in findings across species. Following on from this we discuss VR studies of spatial cognition, outlining limitations and developments, before introducing mobile brain imaging techniques and describe technical challenges and solutions for real-world recording. Finally, we discuss how these advances in mobile brain imaging technology, provide an unprecedented opportunity to illuminate how the brain represents complex multifaceted information during naturalistic navigation.

## Introduction

The ability to navigate is a challenging, high-order cognitive problem. Even in the simplest setting, such as in travel to a landmark in plain sight over open terrain (e.g., towards a church spire), navigating involves determining the required direction of travel and estimating how far to proceed. In reality, however, navigation is rarely this straightforward. Everyday experience typically requires navigation in multiple distinct contexts, varying in time course, familiarity and environmental complexity. For example, walking to work typically involves following a learned route to a known location, whereas walking to a new location for the first time involves mapping out an entirely new route to the goal. In addition to differences in the nature of the navigation required, differences in the properties of the surrounding environment and distance to be covered also exert a defining influence on the combination of cognitive processes involved in navigation (e.g., crossing a toy strewn nursery vs. driving to the airport). Despite the variety and complexity of cognitive processes involved, four decades of electrophysiological and neuroimaging research has successfully identified a number of distinct brain regions that are critical for specific operational aspects of navigation.

A wealth of evidence indicates that hippocampal regions are central to navigation when reaching a goal involves internal representations or “cognitive mapping” of a familiar environment (e.g., O’Keefe and Nadel, 1978; Burgess, 2008; Spiers and Barry, 2015). The parahippocampal cortex and retrosplenial cortex are also known to play distinct roles in navigation, with the former facilitating encoding and recognition of local environmental scenes, and the latter responsible for orientation and direction toward unseen goals in the broader environment (Epstein, 2008; Vann et al., 2009; although see Chadwick and Spiers, 2014). Moreover, it has been proposed that the retrosplenial cortex serves to translate between allocentric (world-referenced) representations in the medial temporal lobe and egocentric (self-referenced) representations in the posterior parietal cortex (Byrne et al., 2007). In addition, the cerebellum is thought to track self-motion (Rochefort et al., 2011), while the pre-frontal cortex is implicated in route planning and is also known to be involved in decision-making and strategy shifts during navigation (Poucet et al., 2004; Spiers, 2008). As will be described below, single-unit electrophysiological work in rodents has also identified brain regions with spatially-tuned firing correlates (for reviews see Taube, 2007; Moser et al., 2008; Derdikman and Moser, 2010; Hartley et al., 2014).

While it is clear that significant progress has been made in identifying the core neural mechanisms involved in navigation, limitations in ecological validity leave important questions unanswered—particularly in relation to the integration of the multifaceted cues required in complex scenarios. In the following section we provide a selective overview of evidence at a cellular level, derived from studies employing lab-based imaging techniques in rodents and humans. After outlining some limitations associated with existing approaches, including recent virtual reality (VR) studies, we highlight developments in mobile imaging technology that provide an exciting opportunity to understand the complexity of real-world navigation in humans.

## Single-Unit Spatial Correlates

**Figure 1:**
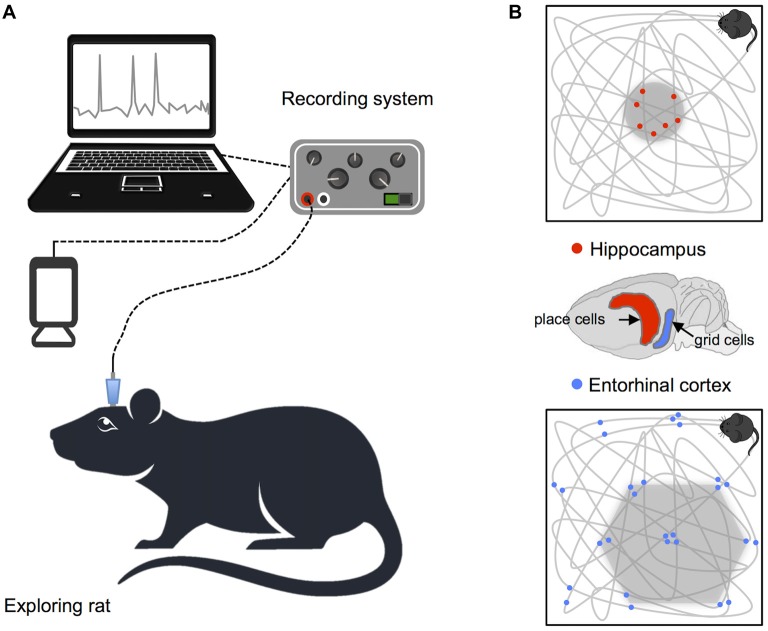
Recording procedure in rodents and basic navigation cells. **(A)** Schematic illustration of the setup for recording single cell data. **(B)** Examples of place and grid cell firing patterns: the gray lines show the animals’ path while exploring a square enclosure and the small dots highlight locations at which single neurons fired action potentials. Brain diagram highlights the location of place cells in the hippocampus and grid cells in the entorhinal cortex.

Studies of animals have led to significant insights regarding how neural systems contribute to identification of spatial location and orientation in the environment. Indeed, the bulk of our current understanding of neural representations of space in the hippocampal formation is derived from single-cell electrophysiological recordings, in rodents, during free movement around purpose built enclosures. In a pioneering study, place cells with spatially specific receptive fields indicating the animal’s current position were identified in CA3 and CA1 regions of the hippocampus (O’Keefe and Dostrovsky, 1971). Since this groundbreaking discovery, subsequent research has identified a number of additional cells that combine to support self-localization and orientation in rodents (see Figure 1). For example, grid cells in the entorhinal cortex code for position, with multiple distributed receptive fields that fire at regularly spaced locations, forming a hexagonal pattern that maps out available space (Fyhn et al., 2004; Hafting et al., 2005). In addition, head direction cells in the limbic system track facing direction, and border cells found in the subiculum, presubiculum, parasubiculum and entorhinal cortex denote proximity to the boundaries of the environment (Taube et al., 1990; Solstad et al., 2008; Lever et al., 2009; Boccara et al., 2010). Equivalent cells supporting spatial representation have also been found in mice and bats (Fyhn et al., 2008; Yartsev et al., 2011; Rubin et al., 2014; Finkelstein et al., 2015) and cells with functions corresponding to place, head direction and grid cells in rodents have also been identified in non-human primates (Robertson et al., 1999; Hori et al., 2003; Killian et al., 2012).

Although a significant amount of research has been done on the single-unit correlates of spatial representations in rodents, the options for using invasive recording techniques in humans is necessarily limited (Hartley et al., 2014). Measurement at the cellular level in humans involves implantation of microelectrodes (depth or surface) and is primarily used to monitor seizure activity in patients suffering from epilepsy. A small number of studies have employed intracranial recording during virtual navigation tasks however, demonstrating the presence of place cells in the hippocampal formation, path cells (tracking direction of movement) in the entorhinal cortex, and grid cells in the cingulate cortex, that resemble those found in rodents (Ekstrom et al., 2003; Jacobs et al., 2010, 2013). Moreover, in both rodents and humans, comparison of movement and slow/still periods has revealed increases in theta during motion (e.g., Ekstrom et al., 2005). These findings are interpreted as evidence of functional parallels between hippocampal oscillations in humans, and those found in rodents during navigation.

While similarities do clearly exist in the basic neural mechanisms implicated in navigation across mammalian species, differences are also apparent, particularly in the nature of theta rhythms. In rodents, active navigation is associated with dominant hippocampal theta oscillations in the 4–10 Hz range, linked to self-motion and temporal coordination of firing patterns in place, grid and head direction cells during navigation (and a wide variety of other behaviors, see Buzsáki, 2005; Jacobs, 2014 for discussion). By contrast, in humans, hippocampal oscillations have been observed at a lower frequency between 1 Hz and 4 Hz (Jacobs et al., 2007), and exhibit shorter bursts than are observed in rodents (Watrous et al., 2011). Moreover, across species differences in the operation of theta rhythms have emerged, which appear to challenge the central role of theta oscillations for human navigation. For example, evidence from non-human primates has revealed variability in the frequency of the dominant theta oscillation (Stewart and Fox, 1991; Skaggs et al., 2007). Additionally, work with bats has demonstrated the operation of grid cells in the entorhinal cortex despite the absence of theta oscillations during active navigation (Yartsev et al., 2011), although this finding has proved somewhat controversial. Crucially, slow movement speed in crawling bats is thought to be responsible for the failure to find the pattern of theta oscillations commonly observed in freely moving rodents, highlighting the importance of matching movement speed in animals (see Barry et al., 2012). This point is not trivial, as evidence also exists of differences in the frequency and duration of navigation-related theta oscillations across species (Watrous et al., 2013), based on recordings in quite different scenarios (i.e., rats completed a Barnes style maze while humans performed a virtual navigation task).

The preceding section emphasizes similarities and differences in the pattern of neural activity underlying navigation across species. Given that the bulk of knowledge about the role of the hippocampal formation in navigation is derived from work with rats, demonstrating parallels across species is valuable. Critically, however, across species neural differences have also been clearly demonstrated. Observations of variability in the underlying frequency profile of hippocampal oscillations across species could be of little functional significance (largely reflecting changes due to neuroanatomical organization or differences in methodology), or they could be interpreted as suggesting that the cognitive and neural processes underlying navigation may not be entirely equivalent in humans and animals. One key issue raised by existing work is the limitation inherent to traditional neuroimaging—whilst rats and other non-humans can be examined during free movement, human studies rarely achieve equivalent realism. The fact that rodent studies reveal specific functional modulations of theta as a consequence of motion illustrates the importance of incorporating movement into human studies. With this in mind, we turn to the use of VR environments for investigating human navigation.

## Virtual Navigation

In recent years, there has been a move toward increasing ecological validity in human imaging studies by employing VR technology to simulate real-world scenarios (Maguire et al., 1997; Riecke et al., 2002; Shelton and Gabrieli, 2002; Spiers and Maguire, 2006). Key strengths of VR include the flexibility and manipulability of the simulated environment, facilitating experimental tasks that are difficult or impossible to implement in real-world settings (whilst also providing a high degree of experimental control, e.g., Kearns et al., 2002). Significant progress in unraveling the complexities of spatial navigation has been made by studies combining fMRI with VR. For example, work to date demonstrates interactions between hippocampal and striatal systems supporting flexible navigation (Brown et al., 2012), the presence of grid-like signals in the entorhinal cortex during VR navigation (Doeller et al., 2010) and imagined navigation (Horner et al., 2016), decoding of goal direction in the entorhinal cortex (Chadwick and Spiers, 2014), and processing of environmental novelty in the hippocampus (Kaplan et al., 2014). In addition, combining MEG and fMRI has revealed memory related increases in theta power during self-initiated movement (by button press) in a VR environment, accompanied by increased activity in the hippocampus (Kaplan et al., 2012). While it is clear that VR combined with static imaging techniques has uncovered a wealth of information, limits on the degree of presence that can be obtained and the absence of idiothetic information may partially disrupt the formation of accurate spatial representations.

**Figure 2:**
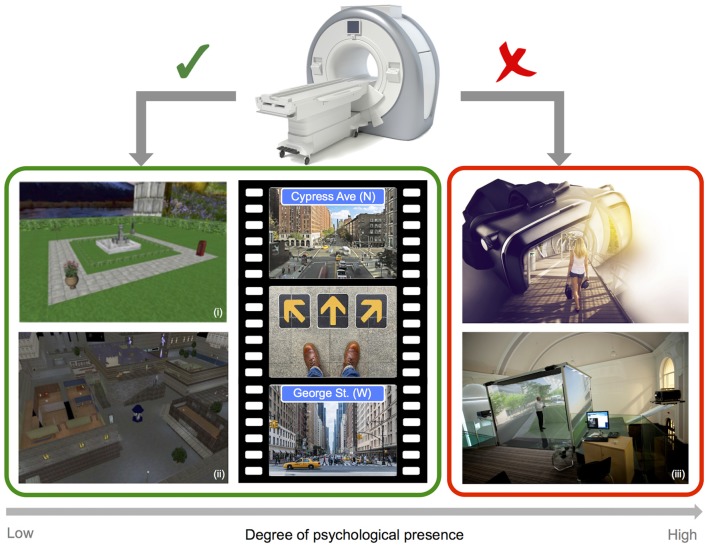
Imaging and virtual reality (VR). Example screenshots of VR style navigation tasks typically employed in the scanner (left), and images of depicting fully immersive 3D VR technology (right), highlighting limitations in the degree of psychological presence that can be obtained when combining a VR approach with static imaging techniques (i: adapted from Chadwick et al., 2015; ii: adapted from King et al., 2005 and iii: Courtesy of Matt Wain photography).

In the case of functional imaging work, scanner limitations impose restrictions on the sorts of VR technology that can be employed, and therefore on the degree of immersion and psychological presence that can be obtained (see Figure 2). In most cases, imaging studies of navigation in humans employ video game style VR displays, where participants control motion through the rendered environment via button presses or joystick input. The approach of combining functional imaging and VR to investigate spatial navigation can be seen as somewhat limited, on the one-hand by the use of 2D displays, and on the other by the absence of combined visual and proprioceptive feedback (Slater et al., 1998). Both of these factors clearly impact the degree of immersion and presence in the virtual environment. Navigating through a VR environment that gives the sense of motion while physically still can result in sensory conflict, and behavioral studies have indicated that this conflict can impair navigation performance when compared to natural walking conditions (Chance et al., 1998; Waller et al., 2004; Ruddle and Lessels, 2006). Importantly, changes in the degree of presence in VR have, in turn, been linked to significant changes in patterns of neural activity. A recent EEG study in humans explicitly compared the effects of 2D (desktop) and 3D (single-wall) interactive VR maze environments. Differences were found in the level and distribution of alpha activity (8–12 Hz) as a function of the degree of reported spatial presence, with greater presence ratings in the 3D environment associated with stronger parietal activation than was observed for the less immersive 2D environment (Kober et al., 2012). While the preceding study investigated spatial presence in VR rather than navigation *per se*, the findings clearly highlight the potential importance of realism for imaging experiments. Importantly, differences in alpha were apparent in posterior parietal regions associated with egocentric representations of the environment in prior fMRI and EEG studies (e.g., Plank et al., 2010; Schindler and Bartels, 2013). Another recent study employing intra-hippocampus EEG recordings during real world and virtual navigation tasks has demonstrated clear differences in theta, with oscillations peaking at a lower frequency band for virtual than for real movement (Bohbot et al., 2017). Moreover, outside of the context of navigation, movement speed has been shown to influence the prevalence of high frequency theta oscillations (6–12 Hz), providing a parallel with prior work in moving rodents (Aghajan et al., 2017). Given that the presence or absence of physical motion systematically alters the pattern of neural activity, it seems clear that the use of static VR approaches to navigation, while undoubtedly valuable, have some limitations.

Ultimately, if different patterns of activation can be observed based on the level of presence and immersion in virtual environments, it is reasonable to believe that neural signals of real-world navigation could also differ from those observed in VR functional imaging studies, particularly when navigation tasks that require updating of orientation and path integration. Efficient updating of egocentric and allocentric spatial representations requires the combination of visual, vestibular and kinesthetic information, with navigators having to adopt different strategies to compensate for the lack of natural movement in static studies (for discussion see Gramann, 2013). Of course, this lack of idiothetic information is not only relevant for studies investigating spatial navigation, but for cognitive neuroscience in general, with evidence supporting embodied accounts of cognition and demonstrating differences in brain states during movement (for discussion see Ladouce et al., 2017). To increase experimental control (e.g., Acharaya et al., 2016) and, in some instances, improve the resolution of neural recordings (Harvey et al., 2009), a number of recent navigation studies have also employed virtual environments with rats, facilitating cross-species comparison. Importantly, this work also highlights that self-motion has a fundamental role in establishing orientation in the environment—modulating navigation related neural patterns. In rats, direct comparison of firing patterns in hippocampal place cells across real and virtual navigation tasks reveals that a high proportion of cells are movement dependent (75%) and also demonstrates an overall reduction in theta power and frequency during static virtual navigation (Chen et al., 2013). Changes in the firing patterns of place cells during random foraging in two-dimensional body-fixed VR have also been observed, with reliance on distal visual cues alone in VR resulting in a marked reduction in spatial selectivity (Aghajan et al., 2015). Similarly, in both humans and nonhuman primates, hippocampal neurons have been found to exhibit weak spatial selectivity when relying on distal visual cues in VR (i.e., in the absence of proximal or vestibular cues; Rolls, 1999; Ekstrom et al., 2003).

Taken together, the evidence demonstrates that static fixed-location VR studies have the advantage of experimental control but do not capture the full sensory richness of actual movement through an environment (for review see Taube et al., 2013). Importantly, this conclusion receives clear support from lesion work in rats, which has independently confirmed the importance of vestibular information for spatial representations (Stackman and Taube, 1997; Stackman et al., 2002). A number of authors have highlighted that although humans have a bias toward the use of visual information (which is inherently easy to manipulate in VR experiments), vestibular, somatosensory, auditory and proprioceptive cues also contribute to navigation (e.g., Brandt et al., 2005; Israël and Warren, 2005; Frissen et al., 2011; Ekstrom et al., 2014; Aghajan et al., 2015; see Taube et al., 2013 for discussion). Theoretically, an important distinction is drawn between two forms of information involved in self-location: sensory perception of environmental features and self-motion information from visual (optic flow), vestibular, proprioceptive and motor systems (Taube et al., 2013; Barry and Burgess, 2014). In reality, however, navigation involves multi-sensory integration with cue salience potentially influencing how cortical hierarchies handle integration of spatially relevant information in any given situation. Ultimately, the broader utility of VR simulations depends upon its ability to accurately represent multiple key features of real-world navigation, including self-motion. Over the last few decades VR technology has developed significantly, such that the most advanced systems can now combine visual, auditory and haptic (tactile) stimulation, while responding to body position and movement using accelerometers, gyroscopes and magnetometers, providing a sense of physical presence and interaction with the virtual environment (Bohil et al., 2011). Indeed, some recent research has focused on investigating spatially relevant cues in VR systems, including visual-vestibular interactions in self-motion (Kim et al., 2015), distance perception (Kelly et al., 2014), and the addition of olfactory cues (Ischer et al., 2014). Overall, most of the evidence to date supports the view that fully immersive 3D VR systems (e.g., CAVE, HMD) can be used to render and manipulate real-world environments reliably for use across a number of applications.

Crucially, movement devices have also been developed to provide a sense of natural movement when exploring VR environments including “VirtuSphere” and the “CyberWalk” omnidirectional treadmill (Hardiess et al., 2015). The “VirtuSphere” is essentially a large-scale hamster ball on a wheeled platform, that the user stands inside wearing a HMD, capable of rolling in any direction based on user input. However, this system has not been widely adopted for research applications to date, and preliminary findings failed to demonstrate an increase in the degree of reported presence, and participants indicated that getting to grips with movement in the sphere was effortful (Skopp et al., 2014). Moreover, work contrasting natural, semi-natural (VirtuSphere) and non-natural (gamepad) locomotion in VR, using path deviation as a measure of accuracy, demonstrated that performance in the VirtuSphere was significantly worse than both other methods of locomotion, which was attributed to significant differences in motions and forces involved in initiating and terminating walking in the VirtuSphere compared to real world walking (Nabiyouni et al., 2015). Omnidirectional treadmill systems like CyberWalk facilitate more natural walking through large scale virtual environments, with evidence indicating that spatial updating performance on the CyberWalk treadmill did not differ from performance for natural walking (Souman et al., 2011). In rats, similar spatial representations (i.e., grid cells, head direction cells and border cells) have also been observed for treadmill navigation that allowed body rotation and real-world navigation, although some differences in the firing pattern of grid cells were observed, with greater spacing in VR than in real-world settings (Aronov and Tank, 2014). In essence, advances in VR technology, including devices used to interact with these systems, make it possible to simulate multiple aspects of real-world navigation. However, for human participants it is not possible to combine high validity VR systems with functional imaging (i.e., fMRI), but recent advances in mobile imaging technology now provide an exciting opportunity to capture neural signals related to navigation during motion.

## Mobile Imaging

Two different technologies facilitating mobile capture of neural activity in real-world contexts have evolved in recent years: EEG and functional Near-Infrared Spectroscopy (fNIRS) systems. EEG is recorded using electrodes placed at specific locations across the surface of the scalp (e.g., frontal, temporal, parietal, occipital etc), and provides a real-time measure of neural activity. EEG cannot be used to investigate deep brain structures, but static EEG has been used in the past to investigate frequency oscillations associated with spatial navigation. For example, work has shown that theta over frontal midline sites is directly related to task difficulty during virtual maze navigation (Bischof and Boulanger, 2003), and to processing of relevant landmarks while navigating in VR (Weidemann et al., 2009; Kober and Neuper, 2011). Using a virtual tunnel navigation task, homing responses consistent with the use of an egocentric reference frame exhibited stronger alpha blocking in the right primary visual cortex, while responses compatible with the use of an allocentric reference frame exhibited stronger alpha blocking in inferior parietal, occipito-temporal and retrosplenial cortices (Gramann et al., 2010b). During path integration in VR adoption of an egocentric reference frame has been associated with modulations of alpha in the parietal, motor and occipital cortices, and theta in the frontal cortices, while adoption of an allocentric reference frame is associated with performance related desynchronization in the 8–13 Hz frequency range and synchronization in the 12–14 Hz range in the retrosplenial complex (Lin et al., 2015). While these static EEG studies are open to the same criticism as the fMRI work outlined above, they clearly demonstrate the utility of EEG to address key questions, highlighting the potential utility of a mobile EEG approach.

Small lightweight battery powered EEG amplifiers were primarily developed for consumer applications (e.g., gaming, ambulatory health monitoring), but recently there has been rapid development of commercial devices better suited to pure research applications (see Park et al., 2015 for discussion of mobile imaging in the context of sports performance). Importantly, recent work contrasting wireless mobile and laboratory based amplifiers reports a high degree of correlation across systems, demonstrating that it is now possible to capture reliable EEG data using mobile technology (De Vos et al., 2014). Moreover, in the last few years there has been a steady growth in the number of studies successfully employing mobile EEG technology to query aspects of cognitive function in real-world contexts (e.g., Gramann et al., 2010a; Wascher et al., 2014). In a recent formative study, mobile EEG was employed to investigate the influence of real-world environments on the formation of episodic memories (Griffiths et al., 2016). Participants were presented with a series of words spaced out along a pre-designated route in an open-field environment, before performing a free recall test. Results replicated subsequent memory effects (contrasting encoding activity for subsequently remembered vs. forgotten items) reported in lab-based studies with power decreases in the low to mid frequency range (<30 Hz), including ubiquitous beta power decreases over the left inferior frontal gyrus and temporal pole regions, providing a clear demonstration that EEG data can be reliably obtained in naturalistic settings. Importantly, the study also set out to investigate the neural correlates of spatial and temporal context clustering. Crucially, this study goes a step beyond merely validating the use of EEG in real-world contexts; it clearly demonstrates the potential utility of a mobile EEG approach, highlighting the importance of environmental context.

Another method that can be used to obtain complimentary information in real-world contexts is fNIRS, which utilizes optical beams of light to monitor cerebral blood flow and hemodynamic response. In basic terms, near-infrared light is beamed onto the surface of the scalp and the level of oxygenation in the underlying region is inferred from the degree of absorption detected as light exits the head, providing an indirect measure of neural activity (Leff et al., 2011). Importantly, comparison with fMRI across a range of cognitive tasks has shown a high correspondence across measures, despite lower spatial resolution in the range of centimeters (Cui et al., 2011). Over the last two decades fNIRS has been applied to a wide range of topic areas, but the bulk of research has still been conducted under laboratory conditions (see Ferrari and Quaresima, 2012 for review). Recently, reports of battery operated wearable/wireless multi-channel systems being used to obtain reliable data in freely moving subjects have begun to emerge, demonstrating that fNIRS is indeed a powerful tool for brain measurement during motion in real-world contexts (e.g., Atsumori et al., 2010; Muehlemann et al., 2013; Piper et al., 2014). Importantly, fNIRS was successfully employed in a recent study contrasting mental workload associated with route following from maps presented on an augmented reality wearable display or a hand-held device during navigation. (McKendrick et al., 2016), providing the first demonstration of the utility of fNIRS for measuring aspects of real world spatial cognition. Moreover, one of the key advantages claimed for fNIRS is the ease of integration with other methods, including EEG (e.g., Chen L. C. et al., 2015; Ahn et al., 2016), making it possible to obtain complimentary spatial and temporal information simultaneosly. While the potential utility of a mobile imaging approach is clear, moving navigation research out of the lab and into the real world raises new challenges that need to be addressed with innovation in experimental design, recording procedures and data analysis.

### Challenges and Solutions

There are a number of challenges associated with recording neural signals in active humans, which have been discussed in depth elsewhere (e.g., see Thompson et al., 2008; Makeig et al., 2009; Gramann et al., 2011, 2014), including handling of motion artifacts, tracking movements in space and synchronizing multiple physiological measures. Over the last decade significant progress has been made, not only in the development of mobile imaging systems, but also in the development of methods for handling the specific challenges presented by recording data during active navigation in real-world contexts. For example, standardized open-source frameworks have been developed which facilitate integration of multiple physiological methods and allow processing and analysis of mobile EEG and body imaging data (e.g., Lab Streaming Layer: Kothe, 2014, MoBILAB: Ojeda et al., 2014; see Reis et al., 2014 for overview of software and hardware solutions). A key problem inherent in imaging cognition in action using EEG is motion artifacts. Traditional cognitive experiments record EEG with participants seated in a dimly lit room, and movement is heavily discouraged to avoid contamination of the neural signal. Time periods of the recording exhibiting strong electrical potentials associated with eye movements and the contraction of muscles are typically rejected or removed using regression procedures in lab-based studies. Advancements in spatial filtering methods such as Independent Components Analysis (ICA) make it possible to isolate motion related artifacts inherent in mobile EEG data. ICA involves linear decomposition of EEG data into independent components and can be implemented to separate brain activity from eye movements, muscle activity and non-brain signals such as line noise (Delorme et al., 2007). Importantly, a number of mobile EEG studies demonstrate that motion artifacts can be adequately addressed using this approach during natural motion and interaction with the environment (e.g., Gwin et al., 2010; Jungnickel and Gramann, 2016; Zink et al., 2016).

Another key problem with conducting mobile experiments in the real-world is establishing the onset of task related cognitive operations. In the lab, computer software is often used to send a TTL pulse to the EEG amplifier signaling the onset of an event of interest, allowing separation of continuous EEG data into time periods associated with different experimental conditions for comparison. At this stage, the majority of mobile EEG studies continue to rely on input from a computer or tablet for accurate timestamping, but recent work has demonstrated that accurate timestamping can be achieved by appeal to concurrently recorded data streams like motion capture (Jungnickel and Gramann, 2016). In addition, an open-source framework has been developed that supports automated identification and labeling of events based on data from recordings of participants performing related tasks using pattern identification (EEG-Annotate: Su et al., 2018). Ultimately, obtaining accurate timestamps in naturalistic settings requires continued innovation, tailored to the specifics of intended experimental design. As adoption of mobile imaging methods grows, development of technical solutions to the unique problems associated with capturing real-world neuroimaging data will continue, but it is clear that significant progress has been made over the last decade in recording and analysis procedures for mobile EEG data and integration with other physiological measures.

Like EEG, fNIRS signal quality can also be impacted by factors relevant for real-world mobile studies, such as motion artifacts, interference from ambient light, sensitivity to changes in temperature and issues with hair (Pringle et al., 1999; McIntosh et al., 2010; Orihuela-Espina et al., 2010; Brigadoi et al., 2014). Recently, innovation in sensor technology has addressed some of these issues, with the development of hair-penetrating brush optodes (Khan et al., 2012) and photodiode sensors that not susceptible to ambient light (for a detailed discussion of hardware see Scholkmann et al., 2014). In the same way as an EEG data recording reflects a combination of activity from brain and non-brain sources, changes in fNIRS signals may not reflect neuronally induced hemodynamic response, instead reflecting task-related changes in systemic activity (e.g., changes in heart rate, blood pressure or breathing rate) or extracerebral hemodynamics (Tachtsidis and Scholkmann, 2016). Recording concurrent physiological measures of systemic activity (e.g., heart rate, respiration, skin conductance) and tracking head motion can assist in separating brain from non-brain sources, by using filtering methods based on PCA and ICA to remove movement-related or physiological artifacts (Herold et al., 2017; see Brigadoi et al., 2014 for a comparison of motion artifact correction methods). As has been noted elsewhere, a more critical limitation of using fNIRS to obtain a direct measure cognitive processing in natural environments is its low temporal resolution: fNIRS has a temporal resolution in the order of seconds, whereas fast cognitive processes occur on a sub-second timescale (e.g., see Gramann et al., 2011). Ultimately scalp EEG and fNIRS cannot be used to investigate deep brain structures (e.g., hippocampus) that have been the focus of the bulk of research in humans employing single-cell recording or fMRI techniques, but obtaining complimentary information that elucidates the spatial and temporal dynamics of real-world navigation is critical. While there are undoubtedly challenges inherent in recording EEG and fNIRS data during motion in real-world contexts, we believe that these issues can and will be adequately addressed over the coming years. Importantly, adoption of a mobile imaging approach for studying navigation should not be seen as a means merely to validate existing findings, but as a complimentary approach that facilitates addressing different sorts of questions about how neural indices of navigation are influenced by dynamic natural environments and physical movement through space.

## The Missing Piece: Moving Humans in Naturalistic Environments

The current review article has highlighted some of the problems that exist for studying human navigation, echoing longstanding concerns that have been expressed about animal navigation, “(…) the role of these various systems can best be understood if one takes into consideration an animal’s behavior in its natural habitat” (Nadel, 1991). As we have described above, studies on the neural systems involved in navigation have primarily been done in either freely-moving rodents or in humans viewing virtual settings while largely immobile. As outlined earlier, self-motion cues are known to be important for navigation tasks involving updating of orientation for path integration and the use of egocentric and allocentric spatial representations. In recent years, there has been an increase in behavioral work querying aspects of navigation using a hybrid VR-real world approach, where natural motion is yoked to movements within virtual environments (e.g., Chen X. et al., 2015; Chen et al., 2017; He and McNamara, 2018; Sjolund et al., 2018), which more closely parallels work carried out with rats. However, this hybrid approach is not generally combined with imaging techniques (although see Ehinger et al., 2014; Gehrke et al., 2018; Liang et al., 2018), so from a theoretical perspective, how the brain integrates sensory perception of environmental features and self-motion information from visual (optic flow), vestibular, proprioceptive and motor systems to support spatial navigation in humans remains an open question. VR will remain a powerful method for studying neural activity during active navigation, as it facilitates manipulations that are difficult or impossible to realize in the real-world. Moreover, combining immersive VR and omnidirectional treadmills with imaging techniques would reflect a significant advancement, particularly in terms of enabling investigation of path integration and reference frames in large scale environments.

Critically, it has been argued that the scale of space employed in common navigation tasks can play a critical role in determining process engagement; limiting inferences that can be drawn from animal studies performed in small-scale space, about neural mechanisms of human spatial navigation in large-scale environments (Wolbers and Wiener, 2014). Prior evidence has also highlighted that a combination of egocentric and allocentric coding strategies is needed to support navigation in large-scale space (Wiener et al., 2013). In essence, mazes employed to investigate navigation in rodents are often simplistic and designed to play to the animals’ strengths (e.g., radial arm, T-maze). By contrast, real world navigation is inherently complex, varying as a function of the availability of visible landmarks or boundaries, and requiring route planning and integration over much larger distances to reach goals that cannot be perceived from the starting location. In addition, in real world environments such as cities, navigating solely on the basis of orientation with respect to the final destination is not always a valuable strategy, and a successful outcome requires greater understanding of how small sub-regions of space link together to create a flexible map of the environment. Successful navigation in real world environments involves appreciation of our own orientation with respect to the surrounding environment, route planning beyond our current field of vision, and constantly updating our frame of reference as we move through the environment towards the goal destination, a situation that is difficult to recreate in the lab.

Our current view of navigation is limited by the simplified conditions that it is typically examined under. In addition to the potential questions highlighted above, regarding the nature of theta rhythms during real-world motion, and the influence of spatial scale on brain activity associated with egocentric and allocentric navigation strategies, the adoption of a mobile approach will open up a range of new questions for investigation. Critically, a mobile approach will provide an unprecedented opportunity for cognitive neuroscientists to investigate how the brain supports interaction with and navigation through dynamic noisy environments, while participating in additional tasks (e.g., walking, talking, driving). How does the brain integrate spatial information from multiple sensory cues and maintain spatial representations in short and long-term memory to support active navigation? How do we plan an optimal route when dealing with multiple destinations? How do the details of the environment (e.g., urban design) influence navigation? Does navigating in a busy city differ from navigating in open space? What happens while interacting with navigation technologies and how do they impact spatial cognition? These are just some examples of the kinds of questions that a mobile imaging approach will enable researchers to address. Importantly, adoption of a mobile approach will undoubtedly improve ecological validity in navigation research, facilitating assessment of natural behavior. Of course, there is a trade-off between ecological validity and experimental control, which will require methodological innovation for progress in investigating real-world aspects of navigation. Ultimately, the real-world cannot be entirely controlled, details of the environment to be navigated will be variable (e.g., temporary landmarks, imposed detours, weather, lighting, traffic, people). However, part of the appeal of a real-world imaging approach lies precisely in understanding how constantly changing environmental factors influence spatial cognition. In this context, methods for adequately monitoring the world, and interactions with it, must be carefully considered during experimental design. Importantly, developments in other technologies such eye-trackers, GPS devices, wearable video recorders, accelerometers, mobile phones and augmented reality wearable displays, make it possible to track relevant behaviors and manipulate details of the natural environment, enabling navigation research to move out of the lab and into the real-world.

## Conclusion

By definition, real-world navigation is complex: multi-sensory inputs, including self-motion cues, are employed to establish an organism’s location in space and to continuously track progress toward a goal location in large scale environments. While mobile EEG and fNIRS cannot provide the spatial resolution of fMRI or the precision of intracranial recording, these approaches can, however, provide data on real-world navigation in humans—an approach to spatial cognition that has not been possible previously. Over the course of this review, we have highlighted the importance of idiothetic cues for orientation, path integration and the use of egocentric and allocentric spatial representations, and as such we believe that examination of these issues will benefit most from adopting a mobile approach. While we highlighted some limitations of static VR approaches, there is no doubt that the development of input devices that support movement is a significant advance. Moreover, we believe that combining fully immersive VR with mobile imaging techniques provides a good balance of ecological validity and experimental control. However, fully immersive VR technology and omnidirectional treadmills are expensive and also require dedicated testing areas and specialist skills to program realistic virtual environments. Moreover, there are some questions that can only be adequately addressed outside of the laboratory. For example, contrasting navigation performance in familiar (e.g., hometown) and unfamiliar environments. At this stage, it remains unclear to what extent current neurobiological models will be supported, augmented or undermined. What is clear, however, is that a move towards mobile imaging will provide a more complete picture, which can fully capture the complexities of real-world navigation.

## Author Contributions

JP performed the reference search and wrote the article. PD and DD provided feedback on drafts and edited the final submission.

## Conflict of Interest Statement

The authors declare that the research was conducted in the absence of any commercial or financial relationships that could be construed as a potential conflict of interest.
